# Gum Arabic Fibers Decreased Inflammatory Markers and Disease Severity Score among Rheumatoid Arthritis Patients, Phase II Trial

**DOI:** 10.1155/2018/4197537

**Published:** 2018-07-05

**Authors:** Ebtihal Kamal, Lamis AbdelGadir Kaddam, Maha Dahawi, Montaser Osman, Mohammed Abdelraman Salih, Alnour Alagib, Amal Saeed

**Affiliations:** ^1^Department of Microbiology and Immunology, Faculty of Medicine, University of Khartoum, Khartoum, Sudan; ^2^Department of Physiology, Faculty of Medicine, Alneelain University Khartoum, Khartoum, Sudan; ^3^Department of Physiology, Faculty of Medicine, University of Khartoum, Khartoum, Sudan; ^4^Department of Clinical Immunology, Sudan Medical Specialization Board, Khartoum, Sudan; ^5^Department of Biochemistry, Faculty of Medicine, University of Khartoum, Khartoum, Sudan; ^6^Department of Rheumatology, Military Hospital, Khartoum, Sudan

## Abstract

**Background:**

Rheumatoid arthritis (RA) is autoimmune inflammatory disease that attacks the synovium of the joints. Both TNF*a* and interleukin-1 play crucial roles in the pathogenesis of RA. Gum Arabic (GA) is gummy exudates from* Acacia senegal* tree. Gum Arabic fermentation by colonic bacteria increases serum butyrate concentrations, so it is considered as prebiotic agent. Gum Arabic (GA) has anti-inflammatory activity through its derivative butyrate. To the best of our knowledge, this is the first study conducted to investigate GA intake on inflammatory markers among RA patients.

**Patients and Methods:**

This is clinical trial phase II in which 40 patients were enrolled aged 18 to 70 years. Patients received 30g/day GA for 12 weeks. TNF *α*, ESR, and complete blood count were measured and DAS-28 was calculated before and after regular GA consumption. Study was approved by the Ethical committee of National Medicines and Poisons Board.

**Results:**

This study showed significant decrease in level of serum TNF *α* (p value 0.05) [95% CI, 0.65 -16.5], ESR (p value 0.011) [95% CI, 2.6 -18.89], and number of swollen and tender joints in RA patients after 12 weeks of GA intake which reflected as significant decrease in disease severity score DAS 28 P.V:0.00 [95% CI, 1.25 -1.99]. On the other hand, GA had trivial change in blood indices.

**Conclusion:**

Gum Arabic has favorable immune modulator effect on rheumatoid arthritis. It can be utilized in clinical practice as adjuvant therapy.

**Trial Registration:**

This trial was registered with ClinicalTrials.gov Identifier: NCT02804581 Registered at 19 June 2016, prospective registration.

## 1. Introduction

Rheumatoid arthritis (RA) is a chronic systemic autoimmune inflammatory disease. In North America and Northern Europe, range from 20 to 50 cases per 100,000 population has been reported while the incidence of RA in developing countries is unknown [1]. RA is chronic relapsing disease that initially attacks the synovium of the joints, with a serious and debilitating sequel. The main pathological feature of RA is inflammation of the synovial membranes (Synovitis),with infiltration of T cells, B cells, macrophages, neutrophils, and synovial fibroblasts into the synovial compartment [2], mainly of diarthrodial joints [3].

Butyrate is a four-carbon fatty acid, which cannot be synthesized by the human cells; it is the end product of colonic bacterial aerobic fermentation of fiber and starch [4]. Butyrate has a potent anti-inflammatory effect. It decreases the proinflammatory cytokine expression through inhibition of NF*κ*B activation which has a pivotal role in mediating many immunological responses, through transcription of inflammatory cytokines, adhesion molecules, chemokines [5], and I*κ*B*α* degradation [5, 6]. Butyrate inhibits TNF *α*–induced expression of vascular cell adhesion molecule-1 (VCAM-1) and intracellular cell adhesion molecule-1 (ICAM-1) in a time- and concentration-dependent manner [7]. Butyrate significantly reduced the stimulated release of IL-5, IL-12, and IL-13. [8]. Also it can suppress the production of IL-2 and IFN-*γ* which are the Th1-associated cytokines [9, 10]. In addition, butyrate is known to act as HDACI in the cells [11, 12]. Dendritic Cells (DC) have the unique property to induce primary immune response, so they are major components of the innate and adaptive immune responses [13]. Suppression of DC and antigen presenting cells (APC) function by butyrate may represent a new, promising approach in immunotherapy [14].

Gum Arabic (GA) is a Ca^2+^-, Mg^2+^-, and K^+^-rich dietary fiber [15]. It is a water-soluble dietary fiber derived from the dried gummy exudates of the stems and branches of* Acacia senegal* [16]. GA modulates immunity in mice [17]. GA was found to increase anti-inflammatory cytokine IL10 and decrease TNF*α* and CRP [18], demonstrating anti-inflammatory effects [17]. GA intake showed reduction in TNF*α* expression in the adipose tissues [19]. Moreover, GA was found to block the hepatic macrophage function [20]. In vitro studies exhibited that GA has a particularly strong effect on IL-10 and IL-6 formation, which are essential regulators of the immune response [13].

We hypothesized Gum Arabic (GA) fermentation delivers short chain fatty acid butyrate, which proved to have modulator properties. The present study tested whether Gum Arabic may influence the clinical course of RA and lower the inflammatory markers.

To the best of our knowledge this is the first study conducted to investigate the effect of oral GA utilization on the level on inflammatory markers among RA patients.

## 2. Patients and Methods

This is a single-armed controlled clinical trial phase II. Total coverage of all rheumatoid arthritis patients attending the referred adult rheumatology clinic in the Military Hospital Khartoum, Sudan, was recruited from June to October 2016. Patients' age ranged between 18 and 70 years. Patients' diagnosis was based on the clinical criteria supported by the laboratory findings (RF &ACCPA +ve). Inclusion criteria were as follows: clinically stable as evidenced by medical history and complete physical examination, including vital signs, performed within a week of the start of the study. Exclusion criteria were as follows: clinically significant abnormal laboratory values for the Complete Blood Count (CBC), Liver Function Test (LFT), and Renal Function Test (RFT). All medications and dosages should be stable for 6 weeks before study entry and continued throughout the duration of the study. Patients with hepatic disease, infectious, or autoimmune liver diseases chronic kidney disease, chronic respiratory diseases, and malignancy were excluded from this study. Patients who developed severe side effects to their current treatments prior to enrollment were also excluded. Patients known to have other associated connective tissue diseases like systemic lupus erythematosus, primary Sjogren syndrome, and systemic sclerosis were excluded. Pregnant ladies were omitted from the study.

### 2.1. Data Collection

All patients were interviewed and examined for gathering of information on disease and treatment history. Disease-related variables included disease onset and duration, presence of extra-articular manifestations, 28 Tender Joint Count (TJC28), and 28 Swollen Joint Count (SJC28) were recorded. Disease activity score (DAS28) is widely used as an indicator of RA disease activity and response to treatment [21, 22]. The joints included in DAS28 in the study were as follows (bilaterally): proximal interphalangeal joints (10 joints), metacarpophalangeal joints (10), wrists (2), elbows (2), shoulders (2), and knees (2). When looking at these joints, both the number of joints with tenderness upon touching (TJC28) and swelling (SJC28) were counted. ESR was measured. The participant asked to make a subjective assessment (visual analog score, VAS) of disease activity during the preceding 7 days on a scale between 0 and 10, where 0 is “no activity” and 10 is “highest activity possible”. With these parameters, DAS28 was calculated by the formula: DAS = 0.56 *∗* √TJC + 0.28 *∗* √SJC + 0.70 *∗* In (ESR) + 0.014 *∗* VAS.

In this study DAS was calculated using the online DAS calculator (http://www.das-score.nl/das28/DAScalculators/dasculators.html). DAS 28 was found to be reliable indicator of disease severity in previous studies [21, 23]. Physical examination was conducted by consultant physician of the unit and registrar in charge.

Body weight and height were measured and the body mass index (BMI = kg/m2) was calculated in all subjects before and after Gum Arabic ingestion.

GA in powder form: it is a 100% natural extract powder produced mechanically from the wildly grown* Acacia senegal* tree with a particle size less than 210 *μ*m with no additives. The dose was 30 grams/day determined based on previous study that revealed 8 weeks of dietary supplementation with 25 g/day of GA would increase twofold in serum butyrate level [24]. Each patient received 28 doses each visit and was constructed to dissolve the powder in 200ml of water and consume it in the early morning. Patients were followed regularly and the following investigations were done during the visit for follow-up: CBC, ESR, LFT, and RFT.

All investigations were performed frequently to monitor the disease activity in response to GA administration. Patients were given contact number and information in case of emergency during the trial.

Three ml of blood was taken in (EDTA) container; CBC was measured by Sysmex KX-21N™ Automated Hematology Analyzer applying electrical impedance and optical method. ESR was measured using Westergren tube. Plasma was separated from EDTA sample and used for measurement of Human TNF*α* level by enzyme-linked immunosorbent assay (ELISA). The commercial kits used were from Biolegend, Ltd., human max standard set.

Our primary outcome was inflammatory markers level and DAS 28 score after 12 weeks of GA oral ingestion.

### 2.2. Statistical Analysis

SPSS 20 was used for data analysis. Paired* t*-test was used to compare between means of different variables before and after GA intake. Repeated-measures analysis of variance (rANOVA) was used to analyze the repeated measures.

### 2.3. Ethical Consideration

Each patient signed a written informed consent; the study was approved by the Ethical committee of National Medicines and Poisons Board. Each patient had the right to discontinue at any time.

This study was registered (prospective registration) at ClinicalTrials.gov ID: NCT02804581

## 3. Results

### 3.1. Patient Enrollment and Study Duration

**Figure 1:**
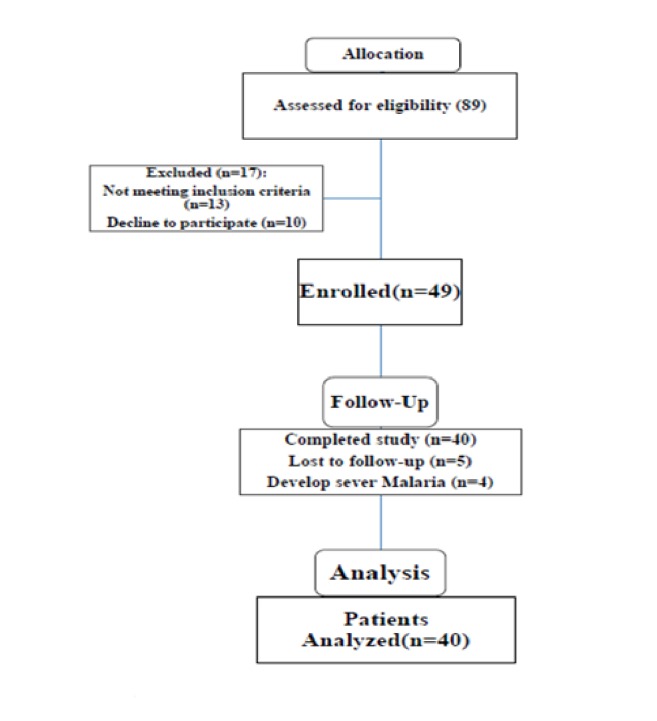
Participant flowchart.

Forty-nine patients were enrolled in this study, from June to October 2016. Duration of treatment was 12 weeks, except three patients who received GA for ten weeks. Nine patients were excluded, five of them were lost during the study period, and four patients developed sever malaria and required admission during the first four weeks of the study. Forty patients completed the study period (Figure 1). All were Sudanese; two were males and the rest were females. The mean age of the female patients was 55±2.8 and mean age of the males was 47.8±12.7. 50% of patients were receiving combination of steroid and hydroxychloroquine, and six patients (15%) combination of steroid, hydroxychloroquine, and methotrexate. Three patients (7.5%) received only steroid and another three patients (7.5%) hydroxychloroquine. The rest of the patients received various combinations of drugs (azathioprine, leflunomide, and sulfasalazine).

**Figure 2:**
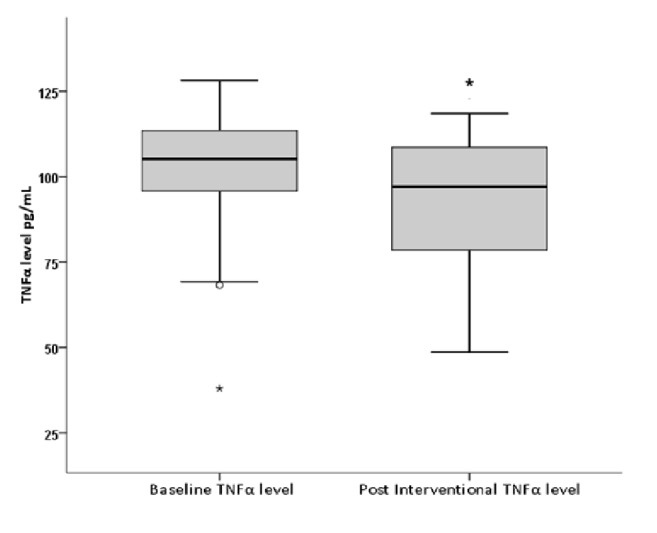
**Effect of Gum Arabic intake on TNF level (P.V=0.05).** *∗* indicates significant difference from baseline.

**Figure 3:**
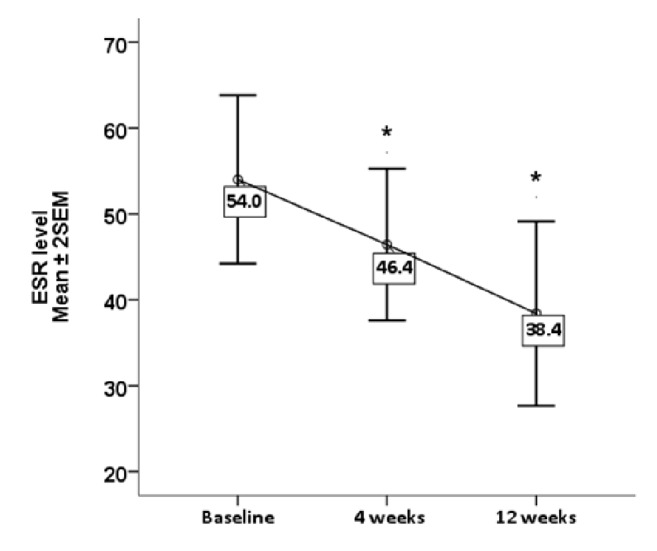
Effect of Gum Arabic intake on ESR Level (P.V=0.011). *∗* indicates significant difference from baseline.

Oral intake of Gum Arabic significantly decreased the level of TNF*α* after Gum Arabic intake (p value 0.05) (Figure 2). ESR level decreased from the first month and was sustained throughout the study (Figure 3). Oral intake of Gum Arabic significantly decreased the SJC, TJC, VAS, and DAS28 (Table 1). There is no significant difference in BMI before and after Gum Arabic intake (p value = 0.107).

Daily intake of Gum Arabic showed no significant change on hemoglobin concentration (P value=0.6). Daily intake of Gum Arabic showed no significant change on MCV, platelet count, or TWBCs count (Table 2).

### 3.2. GA Tolerance and Side Effects

Less than quarter of patients complained from gastrointestinal symptoms, which resolved spontaneously. None of patients stopped GA because of these symptoms.

## 4. Discussion

Forty patients completed the study period. Thirty-eight patients were females (95%). Our results were consistent with previous study which showed increased female-to-male ratio among patients with RA mostly due to changes in the female hormonal level during menstrual cycle, pregnancy, breastfeeding, and the use of the oral contraceptive [25].

TNF*α* is involved in the development of RA, the fact which is supported by successful treatments with anti-TNF*α* factors [11]. According to the present study, GA significantly decreased the level of TNF*α* as shown in Figure 1. Matsumoto's study revealed that ingestion of 25 grams of GA daily doubled serum butyrate level [24]. Butyrate is known to decrease TNF production through NF*κ*B inhibition, as stated in a study done among patients with Crohn disease [6]. Our study revealed a novel effect of GA, as a TNF*α* lowering agent in RA patients. As already known, TNF is a major proinflammatory cytokine implicated in the pathogenesis of RA [26]. Reduction of TNF level resulted in less joints inflammation reflected by reduction in the SJC, TJC, and declined ESR level (inflammatory marker). All of this lead to decrease in DAS 28 value and improvement in patients' clinical condition. In the current study, this was emphasized by improvement in the VAS (Table 2). This was confirmed in this study which revealed significant reduction in ESR (p value=0.011), SJC (p value >0.001), TJC (p value>0.001), VAS (p value>0.001), and DAS (p value> 0.001) as shown in Table 1.

GA intake did not affect hemoglobin concentration and MCV, and there was no significant change on other blood indices as shown in Table 1. Similar result was found in study conducted among sickle cell anemia patients [27, 28]. In contrast to the current result a previous study revealed that GA intake increases MCV [27].

There was no significant difference in platelets count and TWBC before and after GA intake as shown Table 2. This is in agreement with the results of a study done by Kaddam et al. which stated that GA showed no effect on reticulocytes, RBCs, and platelets count with insignificant decrease in WBCs [27]. Another study done by Ross et al. found that there is no effect of GA on the blood indices [29].

GA beneficial effects could be attributed to prebiotic properties. Altered gut microbiota was found to be implicated in RA pathogenesis [30]. Recent studies reveled the substantial role of healthy diet in RA pathogenesis and outcome [31–33].

GA can serve as natural fibers to increase short chain fatty acids level, which yield immunomodulatory effect in favor of reducing inflammation and offering patients' better quality of life.

## 5. Conclusion

In conclusion, Gum Arabic has advantageous immune modulator effect on rheumatoid arthritis. It decreased TNF *α* and ESR level and improved patients' clinical symptoms and signs as it decreases SJC, TJC, VAS, and DAS28. Moreover, GA has no effect on blood indices and BMI.

One of the limitations in our study is not measuring serum butyrate concentration due to resource limitation. Still, our results revealed new approach of RA management that needs more studies. Long duration trial with multiarms will be beneficial to assess the sustainability of the decrease in TNF *α* and generalize the outcomes.

## Figures and Tables

**Table 1:** Comparison between the mean of pre- and post-intervention values of clinical scores.

Variable	Baseline value Mean±SD	Post-intervention value Mean±SD	P.V	95% CI
DAS28	5.43± 1.49	3.8± 1.26	>0.001^**∗∗**^	1.253-1.998
TJC	10.66 ± 9.6	2.97± 6.03	>0.001^**∗∗**^	4.979-10.39
SJC	5.4±6.5	2.05± 4.7	>0.001^**∗∗**^	2.088-4.57

VAS	4.85±2.17	2.1±1.9	>0.001^**∗∗**^	2.19-3.31

^*∗*^ Difference is significant at the 0.05 level (2-tailed).

^*∗∗*^ Difference is significant at the 0.01 level (2-tailed).

**Table 2:** Comparison between the mean of pre- and post-intervention values of hematological indices.

variable	Baseline Mean±SD	12 weeks after GA intake Mean±SD	P.V
Hemoglobin g/dl	12.5±1.1	12.7±1.2	0.6
MCV fl	84.9±6.7	83.5±7.3	0.2
MCH pg	27.5±2.6	27.8±2.7	0.2
Platelets 10^3^ /uL	314±75	325±78	0.2
TWBC10^3^ /uL	7.4±2.4	7.6±2.3	0.3

## Data Availability

All data generated or analyzed during this study are included in this article. Spreadsheet is available upon request from authors.

## List of Abbreviations

CBC: Complete blood count
DAS: Disease activity score
ESR: Erythrocytes sedimentation rate
GA: Gum Arabic
LFT: Liver function test
RA: Rheumatoid arthritis
RFT: Renal function test
SCFA: Short chain fatty acid
SJC: Swollen joint count
TJC: Tender joint count
TNF*α*: Tumor necrosis factor *α*
VAS: Visual analog score.
